# Antineoplastic drugs-related medication-associated pseudoporphyria

**DOI:** 10.1016/j.jdcr.2023.08.019

**Published:** 2023-08-26

**Authors:** Philip R. Cohen

**Affiliations:** a Department of Dermatology, University of California, Davis Medical Center, Sacramento, California; b Touro University California, College of Osteopathic Medicine, Vallejo, California

**Keywords:** antineoplastic, chemotherapy, drug, medication, photoallergic, photosensitivity, phototoxic, prophyria, pseudoporphyria, targeted

*To the Editor:* I read with interest the intriguing report of olaparib-induced pseudoporphyria in a woman with ovarian cancer.^1^ Drug-induced photosensitivity most commonly involves photoallergic and phototoxic reactions.^2^ Medication-associated pseudoporphyria (MAPS) is an uncommon type of drug-induced photosensitivity that has been associated with various agents (Table I)^1^, ^2^, ^3^, ^4^, ^5^, ^6^, ^7^, ^8^, ^9^, ^10^ and most frequently described in patients who have received naproxen.^2^^,^^10^

**Table I:** Drugs that have been observed to cause medication-associated pseudoporphyria∗

Antiarrhythmic Amiodarone
Antifungal Voriconazole
Antihyperglycemic Metformin
Antimicrobials Ampicillin-sulbactum and cefepime Dapsone Fluoroquinolones Nalidixic acid Tetracycline
Antineoplastic agents (see Table III) 5-fluorouracil Flutamide Imatinib mesylate Olaparib Sunitinib
Atypical antipsychotic Olanzapine
Diuretics Butetanide Chlorthalidone Hydrochlorothiazide/triamterene Furosemide Torsemide
Immunosuppressant agent Cyclosporine
Nonsteroidal anti-inflammatory drugs
Aryl propionic acid
Tiaprofenic acid
Cyclooxygenase-1 and cyclooxygenase-2 inhibitor
Mefenamic acid
Cyclooxygenase-2 inhibitor
Celecoxib
Nabumeton
Rofecoxib
Lipoxygenase inhibitor
Benoxaprofen
Priopionic acid derivatives
Ketoprofen
Naproxen
Oxaprozin
Salicylic acid derivative
Aspirin and carisoprodol†
Diflunisal
Oral contraceptive Levonorgestrel and ethinyl estradiol
Retinoids Etretinate Isotretinoin
Vitamin Brewer’s yeast‡ Pyridoxine

∗ Pseudoporphyria has also been associated Coca-Cola excessive consumption, chronic renal failure (in patients receiving hemodialysis or peritoneal dialysis), and ultraviolet light exposure (excessive sun exposure), psoralen and ultraviolet-A treatment, and ultraviolet-A tanning beds.

† The patient received a drug which was a combination of aspirin and carisoprodol; the latter medication is a skeletal muscle relaxant.

‡ This consists of a combination of the following vitamins: B1 (thiamine), B2 (riboflavin), B3 (niacin), B5 (pantothemic acid), B7 (biotin), and B9 (folic acid).

The diagnostic criteria for pseudoporphyria (Table II)^8^, ^9^, ^10^ were originally described in a report on bullous photosensitivity to naproxen in 1990.^10^ More recently, MAPS has been observed in patients receiving antineoplastic treatment Table III.^1^^,^^3^, ^4^, ^5^, ^6^, ^7^ The skin lesions of the woman who was receiving olaparib, a poly-ADP ribose polymerase inhibitor, appeared shortly after starting the drug, persisted while she remained on the drug, and resolved promptly after the drug was discontinued.^1^ The onset of pseudoporphyria was delayed at least several months after initiating the drug in patients receiving tyrosine kinase inhibitors, such as imatinib mesylate for chronic myelogenous leukemia and sunitinib for gastrointestinal stromal tumor and renal cell carcinoma.^6^^,^^7^ MAPS has been observed in men with prostate cancer being treated with the nonsteroidal antiandrogen flutamide.^4^^,^^5^ A single report of a man with metastatic adenocarcinoma of unknown primary developed pseudoporphyria after receiving the thymidylate synthetase inhibitor 5-fluorouracil.^3^

**Table II:** Diagnostic criteria of MAPS∗^,^†

Criteria	Findings
Clinical morphology	Similar to the clinical features of porphyria cutanea tarda. Tense bullae and vesicles and erosions at the sites of prior vesiculobullous skin lesions on sun-exposed skin; lesions most commonly appear on the doral hands; other sites include the fingers, extensor legs, upper chest, or face. Skin fragility, milia, and scarring may also be present on the affected location.
Light microscopy examination	Similar to the findings of porphyria cutanea tarda. A cell-poor noninflammatory subepidermal blister; there may be scant to no lymphocytic perivascular infiltrate. Festooning of the dermal papillae may be present or absent. Periodic acid-Schiff staining shows thickening of the blood vessel walls. Caterpillar bodies may be present; these are linear segmented structures composed of degenerating keratinocytes and basement membrane components that may be seen in the basal layer epidermis overlying the blisters; the bodies stain positive with periodic acid-Schiff stain.
Direct immunofluorescence microscopy examination	Similar to the findings of porphyria cutanea tarda. Granular depositis of IgG and C3 are most commonly present at the basement membrane zone at the dermoepidermal junction and in around the blood vessels in the upper dermis.
Laboratory studies: plasma and urine	In contrast to porphyria cutanea tarda, neither the plasma porphyrin level nor the urine porphrin level is increased.

*C3*, Complement component 3.

∗ The acronym MAPS is derived from the first letter ‘m’ of the word medication, the first letter ‘a’ of the word associated, and the first 2 letters ‘ps’ of the word pseudoporphyria.

† To establish a diagnosis of medication-associated pseudoporphyria, all 4 criteria must be fulfilled. In 1990, indirect immunofluorescence evaluation of serum samples was suggested; these are not routinely performed but may be considered if the differential diagnosis include cell-poor bullous pemphigoid. In 1990, porphyrin studies were recommended of red blood cells; these are not routinely performed during the preliminary evaluation of pseudoporphyria.

**Table III:** Antineoplastic associated MAPS

Drug	MOA	Indication	Comment	Refs
5-fluorouracil	TSI	Breast, colon, rectal, pancreatic, and stomach cancer	A 55-year-old man with metastatic adenocarcinoma of unknown primary. After stopping the drug, blistering did not recur	^3^
Flutamide	NSAA	Prostate cancer	Two men with prostate carcinoma. All of the lesions healed without relapse during 11 months of observation in one of the men who was received the drug for 18 months. Permanent healing resulted after replacing the drug with a different NSSA bicalutamide.	^4^^,^^5^
Imatinib mesylate	TKI	Acute lymphoblastic leukemia, chronic myeloid leukemia, gastrointestinal stromal tumors, and myelodysplastic/myeloproliferative diseases	Seven women and 1 man with chronic myeloid leukemia (8 patients) and acute lymphoblastic leukemia (1 patient). Onset of blisters 8-38 months after starting drug.	^6^
Olaparib	PARPI	Ovarian cancer	A 71-year-old woman with ovarian cancer who blisters began after starting the drug and resolved after stopping the drug.	^1^
Sunitinib	TKI	Gastrointestinal stromal tumor, pancreatic neuroendocrine tumor, and renal cell carcinoma	A 55-year-old man with gastrointestinal stromal tumor and 2 men with renal cell carcinoma. Onset of blisters 4 months-3 years after starting drug.	^7^

*MOA*, Mechanism of action; *NSAA*, nonsteroidal antiandrogen; *PARPI*, poly-ADP ribose polymerase inhibitor; *Refs*, references; *TKI*, tyrosine kinase inhibitor; *TSI*, thymidylate synthetase inhibitor.

In summary, all of the diagnostic criteria for MAP need to be fulfilled to establish the diagnosis.^8^, ^9^, ^10^ MAP is most commonly associated with nonsteroidal antiinflammatory drugs and has been observed in patients receiving numerous classes of drugs.^2^^,^^10^ The development of antineoplastic-related MAPS seems to be increasing.^1^^,^^3^, ^4^, ^5^, ^6^, ^7^ In contrast to the prompt development of MAPS in patients who received 5-fluorouracil, flutamide, and olaparib, there was at at least a 4-month delay in the onset of MAPS-related skin lesions in the patients who were receiving a tyrosine kinase inhibitor.^6^^,^^7^ It is reasonable to speculate that the mechanism of pathogenesis for the development of MAPS is different based on the signaling pathways affected by tyrosine kinase inhibitors.

## Conflicts of interest

Dr Cohen is a consultant for ParaPRO; however, the author has no conflict of interest to declare with regards to this manuscript.

## References

[1] Clark A., Weissman A.S., Crowson A.N., Hirshburg J. (2023). Olaparib-induced pseudoporphyria in a patient with ovarian cancer. JAAD Case Rep.

[2] Stein K.R., Scheinfeld N.S. (2007). Drug-induced phtoallergic and phototoxic reactions. Expert Opin Drug Saf.

[3] Laidman P.J., Gebauer K., Trotter J. (1992). 5-Fluorouracil-induced pseudoporphyria. Aust N Z J Med.

[4] Mantoux F., Bahadoran P., Perrin C., Bermon C., Lacour J.P., Ortonne J.P. (1999). Flutamide–induced late cutaneous pseudoporphyria. Ann Dermatol Venereol.

[5] Borroni G., Brazelli V., Baldini F. (1998). Flutamide-induced pseduoporphyria. Br J Dermatol.

[6] Batrani M., Salhotra M., Kubba A., Agrawal M. (2016). Imatinib mesylate--induced pseudoporphyria in a patient with chronic myeloid leukemia. Indian J Dermatol Venereol Leprol.

[7] Sanz-Motilva V., Martorell-Calatayud A., Llombart B. (2015). Sunitinib-induced pseudoporphyria. J Eur Acad Dermatol Venereol.

[8] Green J.J., Manders S.M. (2001). Pseudoporphyria. J Am Acad Dermatol.

[9] Beer K., Applebaum D., Nousari C. (2014). Pseudoporphyria: discussion of etiologic agents. J Drug Dermatol.

[10] Suarez S.M., Cohen P.R., DeLeo V.A. (1990). Bullous photosensitivity to naproxen: “pseudoporphyria”. Arthritis Rheum.

